# Multicentre cohort study to define and validate pathological assessment of response to neoadjuvant therapy in oesophagogastric adenocarcinoma

**DOI:** 10.1002/bjs.10627

**Published:** 2017-09-25

**Authors:** F Noble, M A Lloyd, R Turkington, E Griffiths, M O'Donovan, J R O'Neill, S Mercer, S L Parsons, R C Fitzgerald, T J Underwood, A Noorani, R Fels Elliott, Z Abdullahi, R de la Rue, J Bornschein, S MacRae, B Nutzinger, N Grehan, G Contino, J Crawte, P A W Edwards, A Miremadi, S Malhotra, A Hayden, R Walker, C Peters, G Hannah, R Hardwick, J Davies, H Ford, D Gilligan, P Safranek, A Hindmarsh, V Sujendran, N Carroll, D McManus, S J Hayes, Y Ang, S R Preston, S Oakes, I Bagwan, R J E Skipworth, V Save, T R Hupp, S Puig, M Bedford, P Taniere, J Whiting, J Byrne, J Kelly, J Owsley, C Crichton, H Barr, N Shepherd, O Old, J Lagergren, J Gossage, A Davies, F Chang, J Zylstra, G Sanders, R Berrisford, C Harden, D Bunting, M Lewis, E Cheong, B Kumar, J H Saunders, I N Soomro, R Vohra, J Duffy, P Kaye, A Grabowska, L Lovat, R Haidry, V Eneh, L Igali, I Welch, M Scott, S Sothi, S Suortamo, S Lishman, D Beardsmore, R Sutaria, M Secrier, M D Eldridge, L Bower, A G Lynch, S Tavaré

**Affiliations:** Cancer Sciences Unit, University of Southampton, Southampton, UK; Centre for Cancer Research and Cell Biology, Queen's University Belfast, Belfast, UK; Department of Surgery, University Hospitals Birmingham NHS Foundation Trust, Birmingham, UK; Hutchison/Medical Research Council Cancer Unit, University of Cambridge, Cambridge, UK; Department of Surgery, Royal Infirmary of Edinburgh, Edinburgh, UK; Department of Surgery, Portsmouth NHS Trust, Portsmouth, UK; Department of Surgery, Nottingham University Hospitals NHS Trust, Nottingham, UK; Medical Research Council Cancer Unit, Hutchison/Medical Research Council Research Centre, University of Cambridge, Cambridge, UK; Department of Histopathology, Cambridge University Hospital NHS Trust, Cambridge, UK; Imperial College, London, UK; Oesophago-Gastric Unit, Addenbrooke's Hospital, Cambridge, UK; Oxford ComLab, University of Oxford, Oxford, UK; Cambridge University Hospitals NHS Foundation Trust, Cambridge, UK; Salford Royal NHS Foundation Trust, Salford; Faculty of Medical and Human Sciences, University of Manchester, Manchester, UK; Salford Royal NHS Foundation Trust, Salford, Leigh NHS Foundation Trust, Wigan; Cancer Research UK Cambridge Institute, University of Cambridge, Cambridge, UK; Royal Surrey County Hospital NHS Foundation Trust, Guildford, UK; Edinburgh Royal Infirmary, Edinburgh, UK; Edinburgh University, Edinburgh, UK; University Hospitals Birmingham NHS Foundation Trust, Birmingham, UK; University Hospital Southampton NHS Foundation Trust, Southampton, UK; Department of Computer Science, University of Oxford, Oxford, UK; Gloucester Royal Hospital, Gloucester, UK; St Thomas's Hospital and King's College London, London, UK; Karolinska Institute, Stockholm, Sweden; Plymouth Hospitals NHS Trust, Plymouth, UK; Norfolk and Norwich University Hospital NHS Foundation Trust, Norwich, UK; Nottingham University Hospitals NHS Trust, Nottingham, UK; University College London, London, UK; Norfolk and Waveney Cellular Pathology Network, Norwich, UK; Wythenshawe Hospital, Manchester, UK; University Hospitals Coventry and Warwickshire NHS Trust, Coventry, UK; Peterborough City Hospital, Peterborough Hospitals NHS Trust, Peterborough, UK; Royal Stoke University Hospital, University Hospitals of North Midlands NHS Trust, Stoke-on-Trent, UK; PortsmouthNHSTrust, Portsmouth, UK

## Abstract

**Background:**

This multicentre cohort study sought to define a robust pathological indicator of clinically meaningful response to neoadjuvant chemotherapy in oesophageal adenocarcinoma.

**Methods:**

A questionnaire was distributed to 11 UK upper gastrointestinal cancer centres to determine the use of assessment of response to neoadjuvant chemotherapy. Records of consecutive patients undergoing oesophagogastric resection at seven centres between January 2000 and December 2013 were reviewed. Pathological response to neoadjuvant chemotherapy was assessed using the Mandard Tumour Regression Grade (TRG) and lymph node downstaging.

**Results:**

TRG (8 of 11 centres) was the most widely used system to assess response to neoadjuvant chemotherapy, but there was discordance on how it was used in practice. Of 1392 patients, 1293 had TRG assessment; data were available for clinical and pathological nodal status (cN and pN) in 981 patients, and TRG, cN and pN in 885. There was a significant difference in survival between responders (TRG 1–2; median overall survival (OS) not reached) and non-responders (TRG 3–5; median OS 2·22 (95 per cent c.i. 1·94 to 2·51) years; *P* < 0·001); the hazard ratio was 2·46 (95 per cent c.i. 1·22 to 4·95; *P* = 0·012). Among local non-responders, the presence of lymph node downstaging was associated with significantly improved OS compared with that of patients without lymph node downstaging (median OS not reached *versus* 1·92 (1·68 to 2·16) years; *P* < 0·001).

**Conclusion:**

A clinically meaningful local response to neoadjuvant chemotherapy was restricted to the small minority of patients (14·8 per cent) with TRG 1–2. Among local non-responders, a subset of patients (21·3 per cent) derived benefit from neoadjuvant chemotherapy by lymph node downstaging and their survival mirrored that of local responders.

## Teaser

Response associated with survival

## Introduction

Neoadjuvant chemotherapy (NAC) followed by surgery, along with perioperative chemotherapy and neoadjuvant chemoradiotherapy, is a standard of care in the management of patients with locally advanced adenocarcinoma of the oesophagus and oesophagogastric junction (OGJ) in the UK^1^. The potential benefits of NAC include: downstaging of the primary tumour^2^ and lymph nodes^3^, increased tumour resectability^4^, elimination of micrometastases^5^ and improved survival^6^. Early assessment of response to NAC may provide information to tailor multimodal therapy^7^.

Both NAC and surgery are associated with considerable morbidity and mortality^8^, and evidence remains inconsistent for the survival benefit for patients who undergo NAC^4,8–9^. The most recent meta-analysis^6^ to compare NAC *versus* surgery alone in 2062 patients suggested a 5·1 per cent absolute survival advantage at 2 years for patients treated with NAC for adenocarcinoma. This is because only a small minority of patients have a significant pathological response to neoadjuvant therapy and it is these patients who gain a significant survival benefit from NAC^10,13^.

There are numerous methods for assessing pathological response to neoadjuvant therapy, but no universal measure is used consistently^10,14–18^. The majority were developed for patients who underwent neoadjuvant chemoradiotherapy and did not differentiate patients based on histology. Few have been validated in patients with oesophageal adenocarcinoma undergoing NAC^2,19–22^. Use of the Tumour Regression Grade (TRG) described by Mandard and colleagues^23^ is suggested by UK guidelines, although this has not gained universal acceptance, and the guidelines give no detail regarding how TRG should be used to guide therapy decisions^1,17^. This system is based on the amount of residual tumour and degree of fibrosis at the primary tumour site, which is graded on a five-point scale^23^. Reports from single-centre cohorts^20,24^ and from small subsets of larger multicentre trials^25^ have identified a significant survival advantage with Mandard TRG 1–2 or TRG 1–3, further confusing clinical decision-making.

A number of clinically important questions could be addressed by a robust and universally accepted measure of response to neoadjuvant treatment, including the development of biomarkers to accurately predict an individual patient's tumour response to preoperative therapy; this would lead to non-responders proceeding directly to surgery or being considered for alternative neoadjuvant regimens, and the identification of patients who are likely to benefit from adjuvant therapy in new stratified trials.

This multicentre cohort study evaluated the current status of neoadjuvant response assessment in multidisciplinary team decision-making via a questionnaire, and aimed to define and validate the pathological assessment of response to NAC in treated oesophagogastric adenocarcinoma. The aim was to provide a consistent, simple, robust and universally acceptable method to assess response to neoadjuvant therapy to allow wider application for both clinical use and biomarker discovery.

## Methods

### Questionnaire

A questionnaire was distributed to 11 upper gastrointestinal cancer centres, all part of the Oesophageal Cancer Clinical and Molecular Stratification (OCCAMS) consortium, to assess the current use of neoadjuvant response assessment in clinical practice (*Appendix S1*, supporting information). The OCCAMS consortium is a UK-wide multicentre consortium to facilitate clinical and molecular stratification of oesophagogastric cancer, with ethical approval for biological sample collection and analysis in conjunction with detailed clinical annotation (Research Ethics Committee number 10/H0305/1).

### Patients

The records of consecutive patients undergoing oesophagogastric resection (tumours of the oesophagus and OGJ only were included) treated at seven centres (University Hospital Southampton NHS Foundation Trust, Belfast Health and Social Care Trust, University Hospitals Birmingham NHS Foundation Trust, University Hospital Cambridge NHS Foundation Trust, Royal Infirmary of Edinburgh, Portsmouth NHS Trust, Nottingham University Hospitals NHS Trust) between January 2000 and December 2013 were reviewed as part of the OCCAMS consortium. All patients were discussed at a specialist multidisciplinary team meeting. Standard staging investigations included high-resolution CT, endoscopic ultrasonography, and latterly integrated fluorodeoxyglucose PET–CT and staging laparoscopy, where indicated. Patients deemed suitable for potential surgical resection with tumours staged as cT2 Nx M0 or cTx N+ M0 were considered for NAC based on local practice and national guidelines^1^.

NAC mainly consisted of cisplatin and 5-fluorouracil (5-FU) (two cycles of cisplatin 80 mg/m^2^ intravenously on day 1 and intravenous infusion of 5-FU 1000 mg/m^2^ over 96 h) or platinum-based triplet therapy. The latter comprised three 21-day cycles of anthracycline, platinum and fluoropyrimidine: ECF (epirubicin 50 mg/m^2^, cisplatin 60 mg/m^2^, both intravenously on day 1 and protracted venous infusion of 5-FU 200 mg/m^2^ per day) or ECX (epirubicin 50 mg/m^2^, cisplatin 60 mg/m^2^, both intravenously on day 1 and capecitabine 625 mg/m^2^ orally twice daily for 21 days) or EOX (epirubicin 50 mg/m^2^ intravenous bolus and oxaliplatin 130 mg/m^2^ intravenous infusion over 2 h on day 1, capecitabine 625 mg/m^2^ orally twice daily for 21 days).

CT or PET–CT was repeated before surgery to assess the response to chemotherapy and disease operability.

Data recorded included demographics, tumour characteristics, resection type and histopathological analysis of the surgical specimen. The TNM classification (7th edition) was used to report tumour stage after analysis of pathology reports^26^. Pathological tumour clearance (R status) was determined according to the Royal College of Pathologists' guidance.

Overall survival (OS) was defined as time from operation to date of death from any cause or date of last review.

### Factors analysed

Pathological response to chemotherapy was assessed using the TRG system, with regression based on the degree of fibrosis and residual cancer cells (TRG 1–5)^23,27^. TRG was scored by specialist gastrointestinal pathologists blinded to the clinical data at the treating cancer centre. Some 10 per cent of cases were validated externally by an independent pathologist as part of the OCCAMS/International Cancer Genome Consortium project^28,29^ with a κ value exceeding 0·8.

All dissected lymph nodes were stained with haematoxylin and eosin, and analysed microscopically for metastatic disease. Lymph node downstaging was defined as any regional lymph node that was positive on clinical evaluation (cN+) which subsequently had no evidence of pathological regional lymph node disease (ypN0), as described previously^24^.

### Statistical analysis

Descriptive data are presented as median (range) unless stated otherwise. Data were analysed using the Kruskal–Wallis, Mann–Whitney *U* and Pearson's χ^2^ tests, as appropriate. Kaplan–Meier analysis, and univariable and multivariable Cox logistic regression modelling were used to assess the relationship between pathological response grading systems and OS. All factors that showed statistical significance in univariable analysis were entered to derive the final model. Stratified analyses were performed based on receipt of NAC, nodal stage and response to chemotherapy. *P* < 0·050 was considered statistically significant for all tests. Statistical analysis was undertaken in SPSS® version 22 (IBM, Armonk, New York, USA).

## Results

### Current clinical use of response assessment to neoadjuvant chemotherapy

The responses from 11 UK cancer centres demonstrated that TRG was the system most widely used to assess response to NAC (used in 8 of 11 centres), and felt to be useful in providing prognostic information for the patient (8 of 11) and making decisions about the modification of adjuvant therapy (9 of 11). There was no consensus on how TRG was being used to influence decision-making for individual patients in practice; the centres used different scores to define responders, with most using TRG 1–3 (5 of 8), and there was a lack of consensus on how adjuvant therapy should be guided by TRG. Some centres would advocate adjuvant therapy based on whether the patient had responded to therapy (5 of 11), whereas others would not use response information (6 of 11) (*Fig. S1*, supporting information).

### Study patients

**Fig. 1:**
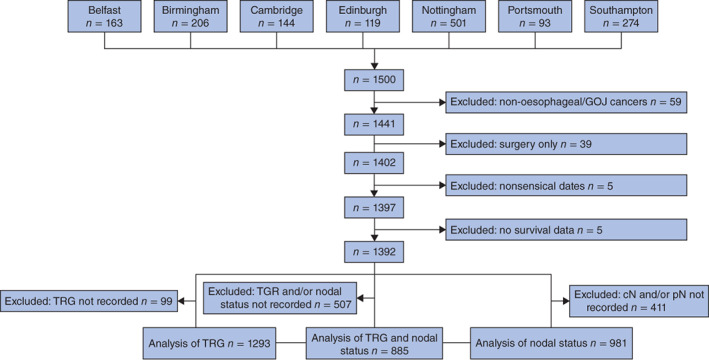
Flow diagram showing numbers of patients contributed by participating centres. GOJ, gastro-oesophageal junction; TRG, Tumour Regression Grade

A total of 1392 patients underwent neoadjuvant therapy with attempted curative resection for oesophageal or OGJ adenocarcinoma, of whom 1293 had TRG assessment. Data were available for both clinical and pathological nodal status (cN and pN) in 981 patients, and TRG, cN and pN status in 885 patients (*Fig. 1*).

Patients were predominantly men (1181 of 1392, 84·8 per cent) and had a median age of 64 (range 26–83) years. Resection clearance (R0) as defined by the Royal College of Pathologists was achieved in 66·6 per cent (913 of 1371) and the median nodal yield was 23 (0–75). Patient characteristics, and clinical and pathological outcomes are summarized in *Table 1*.

**Table 1:** Clinical and pathological characteristics of the full cohort

		No. of patients (*n* = 1392)
Preoperative status		
Age (years)^*^		64 (26–83)
Sex ratio (M : F)		1181 : 211
cT category	cT1	10 (1·0)
	cT2	136 (13·8)
	cT3	798 (80·7)
	cT4	45 (4·6)
	Unknown	403
cN category	cN0	235 (23·8)
	cN1	634 (64·2)
	cN2	102 (10·3)
	cN3	16 (1·6)
	Unknown	405
cM category	cM0	998 (98·7)
	cM1	13 (1·3)
	Unknown	381
Tumour site	Oesophagus	445 (32·0)
	Gastro-oesophageal junction	947 (68·0)
	Siewert 1	290 (42·0)
	Siewert 2	272 (39·3)
	Siewert 3	130 (18·8)
	Siewert unknown	255
Chemotherapy regimen		
	Cisplatin + 5-fluorouracil	281 (20·2)
	Platinum-based triplet therapy	1037 (74·5)
	Other/unknown	74 (5·3)
Pathological outcomes		
ypT category	ypT0	65 (4·7)
	ypT1	135 (9·7)
	ypT2	231 (16·6)
	ypT3	867 (62·4)
	ypT4	91 (6·6)
	Unknown	3
ypN category	ypN0	514 (37·1)
	ypN1	432 (31·2)
	ypN2	246 (17·7)
	ypN3	194 (14·0)
	Unknown	6
ypM category	ypM0	1340 (97·6)
	ypM1	33 (2·4)
	Unknown	19
Tumour response	TRG 1	76 (5·9)
	TRG 2	116 (9·0)
	TRG 3	239 (18·5)
	TRG 4	481 (37·2)
	TRG 5	381 (29·5)
	Unknown	99
Nodal yield^*^		23 (0–75)
% positive nodes^*^		15·6 (0–100)
Patients with lymph nodes downstaged (cN1+ to ypN0)		259 of 981 (26·4)
Resection clearance	R0	913 (66·6)
	R1	458 (33·4)
	Unknown	21
Vascular/lymphatic invasion	Yes	447 (50·2)
	No	443 (49·8)
	Unknown	502
Differentiation	No residual tumour	8 (0·9)
	G1	57 (6·3)
	G2	327 (36·4)
	G3	429 (47·8)
	G4	77 (8·6)
	Unknown	494

Values in parentheses are percentages unless indicated otherwise;

* values are median (range). TRG, Tumour Regression Grade.

Chemotherapy was the predominant neoadjuvant treatment, either platinum-based triplet (1037 of 1392, 74·5 per cent) or cisplatin and 5-FU (281 of 1392, 20·2 per cent). Chemoradiotherapy was used in three patients. In 70·5 per cent of patients (912 of 1293) there were demonstrable signs of local pathological tumour regression (TRG 1–4); 5·9 per cent (76 of 1293) exhibited a complete pathological response (TRG 1). Lymph node downstaging (cN1+ to ypN0) was observed in 26·4 per cent (259 of 981).

### Assessment of clinically meaningful pathological response to neoadjuvant chemotherapy in oesophageal adenocarcinoma

**Fig. 2:**
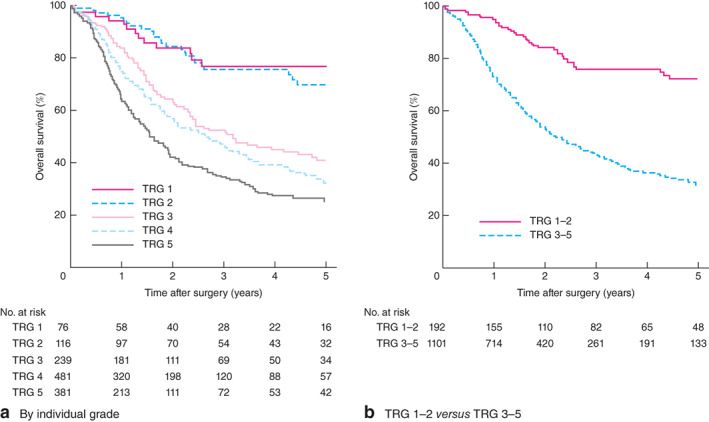
Kaplan–Meier survival analysis of patients grouped according to Tumour Regression Grade (TRG): **a** by individual grade and **b** TRG 1–2 (responders) *versus* TRG 3–5 (non-responders). **a**,**b***P* < 0·001 (log rank test)

Median follow-up for the 1293 patients who underwent NAC with TRG available was 3·6 (95 per cent c.i. 3·2 to 4·1) years. There was a clear association between TRG and prognosis across all groups (*Fig. 2a*, *Table 2*). A significant difference in OS was observed for the 192 patients (14·8 per cent) with TRG 1–2, defined as responders, and the 1101 (85·2 per cent) with TRG 3–5, defined as non-responders (TRG 1–2: median OS not reached, mean OS 7·68 (95 per cent c.i. 7·05 to 8·31) years; TRG 3–5: median OS 2·22 (1·94 to 2·51) years, mean OS 4·06 (3·78 to 4·33) years; *P* < 0·001) (*Fig. 2b*). No significant difference in survival was observed between patients with TRG 1 and those with TRG 2 (median OS not reached in either group; mean OS 7·46 (6·48 to 8·44) *versus* 7·63 (6·84 to 8·43) years; *P* = 0·911).

**Table 2:** Overall survival rates

	No. of patients	Median overall survival	Mean overall survival	*P* ^*^
TRG category				< 0·001
TRG 1	76	n.r.	7·46 (6·48, 8·44)	
TRG 2	116	n.r.	7·63 (6·84, 8·43)	
TRG 3	239	3·19 (2·16, 4·22)	4·90 (4·27, 5·52)	
TRG 4	481	2·64 (2·15, 3·14)	4·16 (3·75, 4·58)	
TRG 5	381	1·57 (1·29, 1·86)	3·39 (2·96, 3·82)	
TRG group				< 0·001
TRG 1–2	192	n.r.	7·68 (7·05, 8·31)	
TRG 3–5	1101	2·22 (1·94, 2·51)	4·06 (3·78, 4·33)	
LN downstaging				< 0·001
LNs downstaged	259	n.r.	7·64 (7·08. 8·20)	
LNs not downstaged	722	2·04 (1·78, 2·30)	3·56 (3·21, 3·99)	
LN downstaging and TRG				< 0·001
TRG 1–2	142	n.r.	7·77 (7·09, 8·45)	
TRG 3–5				
LNs downstaged	158	n.r.	7·24 (6·50, 7·99)	
LNs not downstaged	585	1·92 (1·68, 2·16)	3·29 (2·92, 3·66)	

Values in parentheses are 95 per cent confidence intervals. TRG, Tumour Regression Grade; n.r., not reached; LN, lymph node.

* Log rank test.

Responders and non-responders had similar preoperative clinical features (age, sex) and clinical stage of disease, yet responders had markedly reduced ypT category (*P* < 0·001), ypN status (*P* < 0·001) and were more likely to have nodal downstaging (*P* < 0·001) (*Table 3*). Complete resection (R0) was achieved in 92·5 per cent of responders (173 of 187) compared with 62·5 per cent of non-responders (678 of 1085), and this correlated across the five TRG groups (*Table S1*, supporting information). Of patients who underwent an R1 resection, tumour involvement was at the radial (circumferential) margin in 92·7 per cent, regardless of treatment centre or type of surgery performed. There was no significant difference in nodal yield between responders and non-responders (*P* = 0·437).

**Table 3:** Clinical and pathological characteristics of patients with Tumour Regression Grade available grouped as responders (TRG 1–2) and non-responders (TRG 3–5)

		TRG 1–2 (*n* = 192)	TRG 3–5 (*n* = 1101)	*P* ^†^
Preoperative status				
Age (years)^*^		65 (37–79)	63 (26–83)	0·089^‡^
Sex ratio (M : F)		163 : 29	938 : 163	0·914
cT category	cT1	3 (2·1)	5 (0·7)	0·101
	cT2	26 (18·1)	97 (12·8)	
	cT3	108 (75·0)	625 (82·5)	
	cT4	7 (4·9)	31 (4·1)	
	Unknown	48	343	
cN category	cN0	32 (22·4)	170 (22·7)	0·711
	cN1	96 (67·1)	480 (64·2)	
	cN2	14 (9·8)	84 (11·2)	
	cN3	1 (0·7)	14 (1·9)	
	Unknown	49	353	
cM category	cM0	145 (97·3)	755 (99·0)	0·109
	cM1	4 (2·7)	8 (1·0)	
	Unknown	43	338	
Tumour site	Oesophagus	76 (39·6)	363 (33·0)	0·074
	Gastro-oesophageal junction	116 (60·4)	738 (67·0)	
	Siewert 1	35 (40)	224 (43·8)	0·617
	Siewert 2	33 (38)	198 (38·7)	
	Siewert 3	19 (22)	90 (17·6)	
	Siewert unknown	29	226	
Chemotherapy regimen				
	Cisplatin + 5-fluorouracil	14 (7·3)	236 (21·4)	< 0·001
	Platinum-based triplet therapy	160 (83·3)	815 (74·0)	
	Other/unknown	18 (9·4)	50 (4·5)	
Pathological outcomes				
ypT category	ypT0	64 (33·7)	0 (0)	< 0·001
	ypT1	49 (25·8)	74 (6·7)	
	ypT2	33 (17·4)	176 (16·0)	
	ypT3	42 (22·1)	770 (70·0)	
	ypT4	2 (1·1)	80 (7·3)	
	Unknown	2	1	
ypN category	ypN0	145 (75·9)	336 (30·7)	< 0·001
	ypN1	33 (17·3)	372 (33·9)	
	ypN2	12 (6·3)	213 (19·4)	
	ypN3	1 (0·5)	175 (16·0)	
	Unknown	1	5	
ypM category	ypM0	189 (99·0)	1058 (97·3)	0·179
	ypM1	2 (1·0)	29 (2·7)	
	Unknown	1	14	
Nodal yield^*^		22 (3–65)	23 (0–75)	0·437^‡^
% positive nodes^*^		2·9 (0–54·2)	17·9 (0–100)	< 0·001^‡^
Patients with lymph nodes downstaged (cN1+ to ypN0)		85 of 142 (59·9)	158 of 742 (21·3)	< 0·001
Resection clearance	R0	173 (92·5)	678 (62·5)	< 0·001
	R1	14 (7·5)	407 (37·5)	
	Unknown	5	16	
Vascular/lymphatic invasion	Yes	15 (11·6) 114 (88·4)	364 (54·9) 299 (45·1)	< 0·001
	No	63	438	
	Unknown	8 (7·8)	0 (0)	< 0·001
Differentiation	No residual tumour	20 (19·4)	34 (4·9)	
	G1	37 (35·9)	263 (37·7)	
	G2	34 (33·0)	332 (47·6)	
	G3	4 (3·9)	69 (9·9)	
	G4	89	403	

Values in parentheses are percentages unless indicated otherwise;

* values are median (range). TRG, Tumour Regression Grade.

† Pearson's χ^2^ test, except

‡ Mann–Whitney *U* test.

**Fig. 3:**
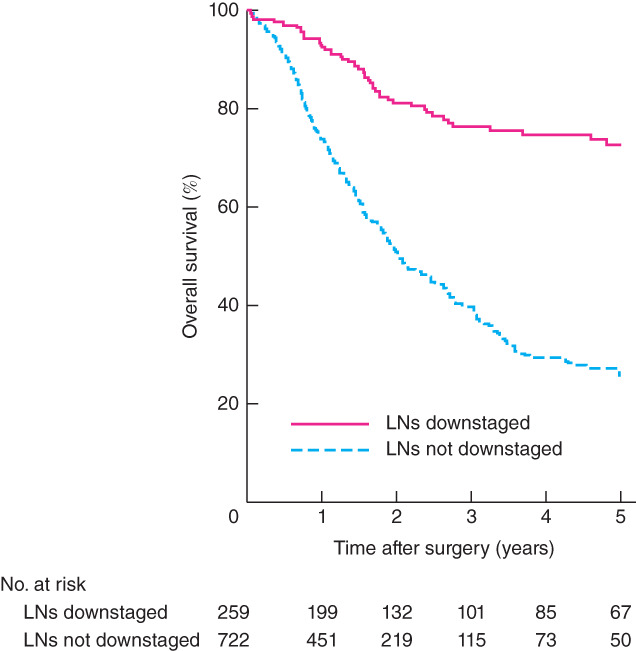
Kaplan–Meier survival analysis of patients grouped according to lymph node (LN) downstaging. *P* < 0·001 (log rank test)

Patients with lymph node downstaging following NAC (259 of 981) had improved OS compared with patients without downstaging (lymph nodes downstaged: median OS not reached, mean OS 7·64 (95 per cent c.i. 7·08 to 8·20) years; lymph nodes not downstaged: median OS 2·04 (1·78 to 2·30) years, mean OS 3·56 (3·21 to 3·99) years; *P* < 0·001) (*Fig. 3*).

Univariable and multivariable analysis confirmed known predictors of OS in oesophageal adenocarcinoma (*Table 4*). Factors that retained significance for the prediction of worse OS in multivariable analysis were: vascular/lymphatic invasion (hazard ratio (HR) 1·61, 95 per cent c.i. 1·23 to 2·10; *P* < 0·001), lack of significant response to NAC (TRG 3–5) (HR 2·46, 1·22 to 4·95; *P* = 0·012), and ypN and ypM status.

**Table 4:** Univariable and multivariable Cox regression analysis of patient, treatment and tumour factors associated with overall survival for patients who received neoadjuvant chemotherapy

	Univariable analysis		Multivariable analysis	
	Hazard ratio	*P*	Hazard ratio	*P*
Patient factors				
Age	1·00 (0·99, 1·01)	0·918		
Sex				
F	1·00 (reference)			
M	1·08 (0·88, 1·34)	0·458		
Chemotherapy regimen				
Platinum-based triplet therapy	1·00 (reference)		1·00 (reference)	
Cisplatin + 5-fluorouracil	1·44 (1·22, 1·70)	< 0·001	1·07 (0·82, 1·41)	0·610
Tumour response				
TRG category				
TRG 1	1·00 (reference)			
TRG 2	1·03 (0·54, 1·98)	0·932		
TRG 3	2·80 (1·61, 4·89)	< 0·001		
TRG 4	3·50 (2·04, 5·99)	< 0·001		
TRG 5	4·81 (2·81, 8·25)	< 0·001		
TRG group				
TRG 1–2	1·00 (reference)		1·00 (reference)	
TRG 3–5	3·66 (2·65, 5·06)	< 0·001	2·46 (1·22, 4·95)	0·012
Lymph nodes downstaged				
Yes	1·00 (reference)		1·00 (reference)	
No	3·99 (2·98, 5·34)	< 0·001	1·59 (0·85, 2·99)	0·149
Tumour factors				
ypT category				
ypT0	1·00 (reference)		1·00 (reference)	
ypT1	1·41 (0·69, 2·87)	0·343	0·59 (0·12, 2·88)	0·510
ypT2	2·38 (1·24, 4·57)	0·009	0·49 (0·10, 2·38)	0·378
ypT3	5·00 (2·67, 9·34)	< 0·001	0·67 (0·14, 3·25)	0·623
ypT4	8·55 (4·35, 16·79)	< 0·001	0·94 (0·18, 4·80)	0·937
ypN category				
ypN0	1·00 (reference)		1·00 (reference)	
ypN1	2·78 (2·26, 3·43)	< 0·001	1·86 (1·04, 3·34)	0·038
ypN2	3·85 (3·04, 4·86)	< 0·001	2·50 (1·38, 4·51)	0·002
ypN3	7·72 (6·08, 9·81)	< 0·001	4·30 (2·36, 7·84)	< 0·001
ypM category				
ypM0	1·00 (reference)		1·00 (reference)	
ypM1	3·05 (2·10, 4·43)	< 0·001	2·51 (1·49, 4·25)	0·001
Vascular/lymphatic invasion				
No	1·00 (reference)		1·00 (reference)	
Yes	2·88 (2·33, 3·56)	< 0·001	1·61 (1·23, 2·10)	< 0·001
Resection margin				
R0	1·00 (reference)		1·00 (reference)	
R1	2·23 (1·98, 2·67)	< 0·001	1·26 (0·97, 1·63)	0·086
Differentiation				
G1	1·00 (reference)		1·00 (reference)	
G2	1·71 (1·03, 2·85)	0·038	0·96 (0·52, 1·78)	0·888
G3	2·76 (1·68, 4·52)	< 0·001	1·08 (0·58, 1·99)	0·814
G4	2·71 (1·55, 4·76)	< 0·001	0·89 (0·43, 1·81)	0·742

Values in parentheses are 95 per cent confidence intervals. TRG, Tumour Regression Grade.

### Evaluation of chemotherapy regimen

Patients treated with platinum-based triplet chemotherapy had a significantly greater response to chemotherapy in the local tumour (TRG) and regional lymph nodes, and were more likely to have an R0 surgical resection than those who received cisplatin and 5-FU (*Table 3*; *Table S2*, supporting information).

Kaplan–Meier analysis showed better OS among patients who had platinum-based triplet chemotherapy (*Fig. S2*, supporting information), but multivariable analysis showed no difference in OS between regimens (*Table 4*).

### Evaluation of combined local tumour response and lymph node downstaging

**Fig. 4:**
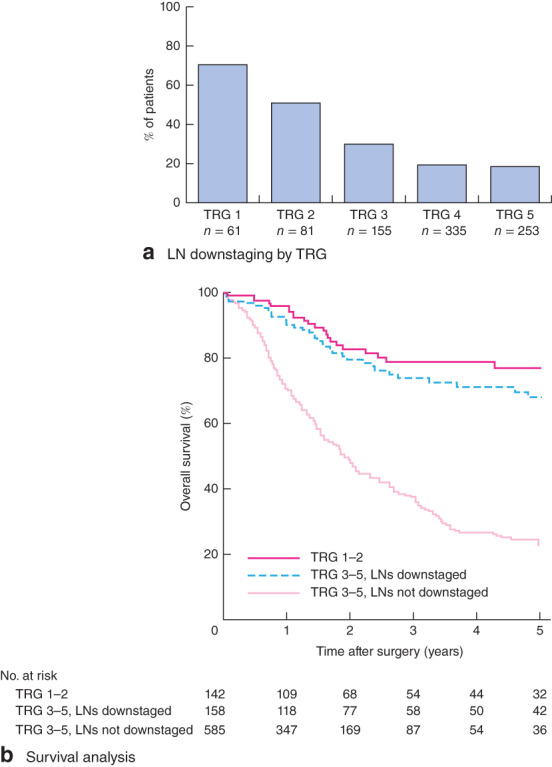
Effect of lymph node (LN) downstaging on survival. **a** Percentage of patients with LN downstaging grouped by Tumour Regression Grade (TRG). **b** Kaplan–Meier curves for patients with local tumour response (TRG 1–2) compared with non-responders (TRG 3–5) divided into those with evidence of LN downstaging or no downstaging. *P* < 0·001 (log rank test)

Some 85 (59·9 per cent) of the 142 patients with a local response to NAC (TRG 1–2) also had regional lymph nodes downstaged, compared with only 158 (21·3 per cent) of 743 non-responders (TRG 3–5) (*P* < 0·001) (*Fig. 4*).

Lymph node downstaging in local non-responders (TRG 3–5) was associated with significantly improved OS (lymph nodes downstaged: median OS not reached, mean OS 7·24 (95 per cent c.i. 6·50 to 7·99) years; lymph nodes not downstaged: median OS 1·92 (1·68 to 2·16) years, mean OS 3·29 (2·92 to 3·66) years; *P* < 0·001).

## Discussion

In this multicentre study, a clinically meaningful local response to NAC for adenocarcinomas of the oesophagus and OGJ was restricted to the small minority of patients (14·8 per cent) with TRG 1–2. Among apparent local non-responders, there was a subset of patients who appeared to derive additional benefit from NAC by lymph node downstaging and their survival mirrored that of local responders.

The difficulties faced by clinicians in routine practice regarding what constitutes a meaningful response to NAC, and how this information should be used to tailor subsequent treatment, has been caused primarily by the cohort sizes of previous studies. For example, the Mandard system was developed in 1994 in a cohort of 93 French patients (84 per cent with squamous cell cancer) treated with cisplatin and radiotherapy, and TRG 1–3 was found to correlate with improved disease-free survival^23^. In a subsequent study performed in the UK and Ireland^30^, TRG had no correlation with survival in 43 patients with adenocarcinoma. More recent work from single institutions has demonstrated the validity of the TRG system in patients with oesophageal cancer treated with NAC, but cohort sizes remain small (Fareed *et al*.^19^: 103 patients, TRG 1–3 associated with a disease-specific survival advantage; Noble *et al*.^24^: 136 patients, TRG 1–2 associated with a disease-free survival advantage). Larger series have focused on the role of NAC and tumour stage rather than TRG^31^, or have included mainly gastric cancers^25^. In the context of neoadjuvant chemoradiotherapy a three-point scale for TRG, using TRG 1 compared with TRG 2–3 and TRG 4–5, was found to provide the best discriminant fit of all response measurement modalities in a cohort of 393 patients from a single centre in Ireland^32^. The data presented here do not support this classification, and suggest that there may be differences in the histomorphological assessment of response between chemotherapy and chemoradiation.

The strengths of the present study are its cohort size, length of follow-up and multicentre nature. It shows that TRG is a robust measure of local response to NAC in routine clinical practice, with excellent correlation between local centre scoring and central validation. The inclusion of the two UK centres (Nottingham^19^ and Southampton^24^) that previously published discordant results regarding the level of TRG associated with a response adds weight to the finding that only TRG 1–2 represents a group of true local responders. This is supported by the use of OS as the outcome measure here, and by the similarity between responder and non-responder groups in terms of pretreatment characteristics.

In contrast to the results of a recent subgroup analysis of the Medical Research Council (MRC) Adjuvant Gastric Infusional Chemotherapy (MAGIC) Trial^25^, where lymph node status was the only independent predictor of survival in patients treated with chemotherapy, in the present study non-response to NAC (TRG 3–5) was independently associated with poor overall survival. This probably reflects the relative sizes of the study cohorts (1293 in the present study *versus* 330 in MAGIC^25^). In the present study, no attempt was made to assess the value of postoperative chemotherapy in either responders or non-responders. In a recent study of 333 patients from a single UK centre^33^, only responders to NAC were observed to derive a survival advantage from the adjuvant part of the MAGIC regimen, and there was evidence of potential harm for non-responders in terms of chemotherapy morbidity. The question of who should receive adjuvant treatment and what form this should take needs to be addressed urgently in prospective studies, so that futile overtreatment with chemotherapy can be avoided and therapies can be targeted more effectively where appropriate. TRG and lymph node downstaging could be used to stratify patients while the validation of recently discovered mutational endotypes takes place^28^.

This study was not designed to investigate differences in outcome between different chemotherapy regimens. However, the data clearly show the superiority of platinum-based triplet chemotherapy, including epirubicin, over cisplatin and 5-FU for local tumour response, but this did not translate into better survival. These findings are in keeping with the results of the MRC OEO5 trial^34^, in which two cycles of neoadjuvant cisplatin and 5-FU was shown to be equivalent to four cycles of ECX for overall survival, with higher chemotherapy-related toxicity in the ECX group^34^. Widely regarded as a negative trial, OEO5 is important because it identifies the requirement for robust markers of patient and tumour stratification to guide precision treatment.

There are clear drawbacks when performing a large multicentre cohort study over a relatively long period. This was not a randomized trial and there are missing data, so bias cannot be excluded, but the sample size helps to negate this deficiency. Staging modalities, chemotherapy and to a lesser extent surgery will have changed over the study interval. It is possible that a number of patients who received treatment at the beginning of the study may have been excluded from treatment had they been staged using modern imaging, leading to worsened OS. It could be presumed that these patients would be in the non-responder group, as there is some evidence to support the association between local tumour response and systemic relapse, but tumour stage after NAC seems to be more important than initial stage at presentation in terms of assessing prognosis^31^. These drawbacks may also explain the relatively low overall R0 resection rate (66·6 per cent), but it is important to note that the circumferential margin was involved in the majority of R1 resections and the more stringent Royal College of Pathologists' definition of margin involvement was used. No attempt has been made to assess the theoretical benefit of using NAC (rather than neoadjuvant chemoradiotherapy) to treat distant micrometastatic disease in these patients. Ongoing randomized studies will hopefully answer this important question.

The finding that a small group (approximately 20 per cent) of patients whose primary tumours apparently do not respond to NAC have downstaging of local nodes and associated good long-term survival, similar to that of primary tumour responders, is important both for discussions of prognosis with individual patients and for the design of the next generation of trials of tailored adjuvant treatment. The use of cN category to assess involvement of nodes before operation compared with postoperative ypN status to determine downstaging is open to criticism. In support of this strategy, it has been shown previously that patients with ypN0 disease have worse OS than those with pN0 disease, suggesting true nodal involvement^24^, and downstaging has been demonstrated accurately in other cohorts^31^. This also reflects current clinical practice in the UK and elsewhere. However, an alternative interpretation of these data is that they are indicative of clinical overstaging rather than downstaging. A future analysis should consider the pathological assessment of nodal downstaging to look for evidence of fibrosis/previous tumour in the nodes, and the relationship between this and prognosis. Large-scale collaborations such as the OCCAMS consortium are ideally placed to do this.

As the research community begins to consider the move from binary ‘one size fits all’ treatment and trial designs to more personalized strategies, robust markers of treatment response will be required. The present findings confirm TRG as such a marker and clearly define groups of patients who benefit from NAC. In addition, giving clarity to the assessment of response offers the opportunity to determine biomarkers that may predict response to existing and novel neoadjuvant treatments, whether they are patient-, tumour- or treatment-related.

## Collaborators

OCCAMS consortium collaborators are: A. Noorani, R. Fels Elliott, Z. Abdullahi, R. de la Rue, J. Bornschein, S. MacRae, B. Nutzinger, N. Grehan, G. Contino, J. Crawte, P. A. W. Edwards (Medical Research Council Cancer Unit, Hutchison/Medical Research Council Research Centre, University of Cambridge, Cambridge, UK); A. Miremadi, S. Malhotra (Department of Histopathology, Cambridge University Hospital NHS Trust, Cambridge, UK); A. Hayden, R. Walker (Cancer Sciences Unit, University of Southampton, Southampton, UK); C. Peters, G. Hannah (Imperial College, London, UK); R. Hardwick (Oesophago-Gastric Unit, Addenbrooke's Hospital, Cambridge, UK); J. Davies (Oxford ComLab, University of Oxford, Oxford, UK); H. Ford, D. Gilligan, P. Safranek, A. Hindmarsh, V. Sujendran, N. Carroll (Cambridge University Hospitals NHS Foundation Trust, Cambridge, UK); D. McManus (Centre for Cancer Research and Cell Biology, Queen's University Belfast, Belfast, UK); S. J. Hayes (Salford Royal NHS Foundation Trust, Salford, and Faculty of Medical and Human Sciences, University of Manchester, Manchester, UK); Y. Ang (Salford Royal NHS Foundation Trust, Salford, Leigh NHS Foundation Trust, Wigan, and Cancer Research UK Cambridge Institute, University of Cambridge, Cambridge, UK); S. R. Preston, S. Oakes, I. Bagwan (Royal Surrey County Hospital NHS Foundation Trust, Guildford, UK); R. J. E. Skipworth, V. Save (Edinburgh Royal Infirmary, Edinburgh, UK); T. R. Hupp (Edinburgh University, Edinburgh, UK); S. Puig, M. Bedford, P. Taniere, J. Whiting (University Hospitals Birmingham NHS Foundation Trust, Birmingham, UK); J. Byrne, J. Kelly, J. Owsley (University Hospital Southampton NHS Foundation Trust, Southampton, UK); C. Crichton (Department of Computer Science, University of Oxford, Oxford, UK); H. Barr, N. Shepherd, O. Old (Gloucester Royal Hospital, Gloucester, UK); J. Lagergren (St Thomas's Hospital and King's College London, London, UK, and Karolinska Institute, Stockholm, Sweden); J. Gossage, A. Davies, F. Chang, J. Zylstra (St Thomas's Hospital and King's College London, London, UK); G. Sanders, R. Berrisford, C. Harden, D. Bunting (Plymouth Hospitals NHS Trust, Plymouth, UK); M. Lewis, E. Cheong, B. Kumar (Norfolk and Norwich University Hospital NHS Foundation Trust, Norwich, UK); J. H. Saunders, I. N. Soomro, R. Vohra, J. Duffy, P. Kaye, A. Grabowska (Nottingham University Hospitals NHS Trust, Nottingham, UK); L. Lovat, R. Haidry, V. Eneh (University College London, London, UK); L. Igali (Norfolk and Waveney Cellular Pathology Network, Norwich, UK); I. Welch, M. Scott (Wythenshawe Hospital, Manchester, UK); S. Sothi, S. Suortamo (University Hospitals Coventry and Warwickshire NHS Trust, Coventry, UK); S. Lishman (Peterborough City Hospital, Peterborough Hospitals NHS Trust, Peterborough, UK); D. Beardsmore (Royal Stoke University Hospital, University Hospitals of North Midlands NHS Trust, Stoke-on-Trent, UK); R. Sutaria (Portsmouth NHS Trust, Portsmouth, UK); M. Secrier, M. D. Eldridge, L. Bower, A. G. Lynch, S. Tavaré (Cancer Research UK Cambridge Institute, University of Cambridge, Cambridge, UK).

## Supplementary Material

bjs10627-0001-AppendixS1 **Appendix S1** Site-specific questionnaire

bjs10627-0002-FigureS1 **Fig. S1** Responses to questionnaire sent to 11 UK cancer centres to determine current use of pathological response information in clinical decision-making

bjs10627-0003-FigureS2 **Fig. S2** Kaplan–Meier curves for patients treated with cisplatin and 5-fluorouracil or platinum-based triplet chemotherapy

bjs10627-0004-TableS1 **Table S1** Resection margin involvement in relation to tumour regression grade (Word document)

bjs10627-0005-TableS2 **Table S2** Effect of chemotherapy regimen on tumour regression grade, lymph node downstaging and resection margins
