# Supplementary material for: Multicentre cohort study to define and validate pathological assessment of response to neoadjuvant therapy in oesophagogastric adenocarcinoma
Source: Br J Surg. 2017 Sep 25;104(13):1816–28. doi: 10.1002/bjs.10627 (PMC5725679; doi:10.1002/bjs.10627)

## BJS10627

## Multicentre cohort study to define and validate pathological assessment of response to neoadjuvant therapy in oesophagogastric adenocarcinoma

F. Noble, M. A. Lloyd, R. Turkington, E. Griffiths, M. O’Donovan, J. R. O’Neill, S. Mercer^6^, S. L. Parsons, R. C. Fitzgerald and T. J. Underwood, on behalf of the OCCAMS consortium

## Appendix S1 Site-specific questionnaire

| **SITE SPECIFIC QUESTIONNAIRE (SSQ)**  **(Circle/Delete as appropriate)** | | | | |
| --- | --- | --- | --- | --- |
| HOSPITAL NAME |  | | | |
| OCCAMS LEAD |  | | | |
| Centre Collaborators |  | | | |
| Is Response to Neoadjuvant therapy used in patient management at your multidisciplinary team meeting (MDT)? | | Yes | No | |
| What Response criteria do you use? | RECIST | Mandard (TRG) | Lymph node (LN) downstaging | Other  ……… |
| What are the pathological response to neoadjuvant therapy scoring systems used for by your MDT? | TRG | Prognostic information for the patient? | Yes | No |
|  |  | Prognostic information for the clinician? | Yes | No |
|  |  | Inform the use of Adjuvant therapies? | Yes | No |
|  | Other  ……….. | Prognostic information for the patient? | Yes | No |
|  |  | Prognostic information for the clinician? | Yes | No |
|  |  | Inform the use of Adjuvant therapies? | Yes | No |
|  | Other  ……….. | Prognostic information for the patient? | Yes | No |
|  |  | Prognostic information for the clinician? | Yes | No |
|  |  | Inform the use of Adjuvant therapies? | Yes | No |
| How does your MDT define a significant response to neoadjuvant therapy using the Mandard scoring system? | | Complete pathological response (pCR, TRG 1)? | Yes | No |
|  |  | TRG 1-2 | Yes | No |
|  |  | TRG 1-3 | Yes | No |
|  |  | Other |  | |
| If your MDT uses pathological response to neoadjuvant therapy to inform their decision in offering adjuvant therapy which patients are most likely to be offered adjuvant therapy? | | Responders | Non-Responders | |
|  |  | Other  ……………………………………………………. | | |
| Will alternative oncological regimes be offered to patients who are non-responders to neoadjuvant therapy? | | Yes | No | |


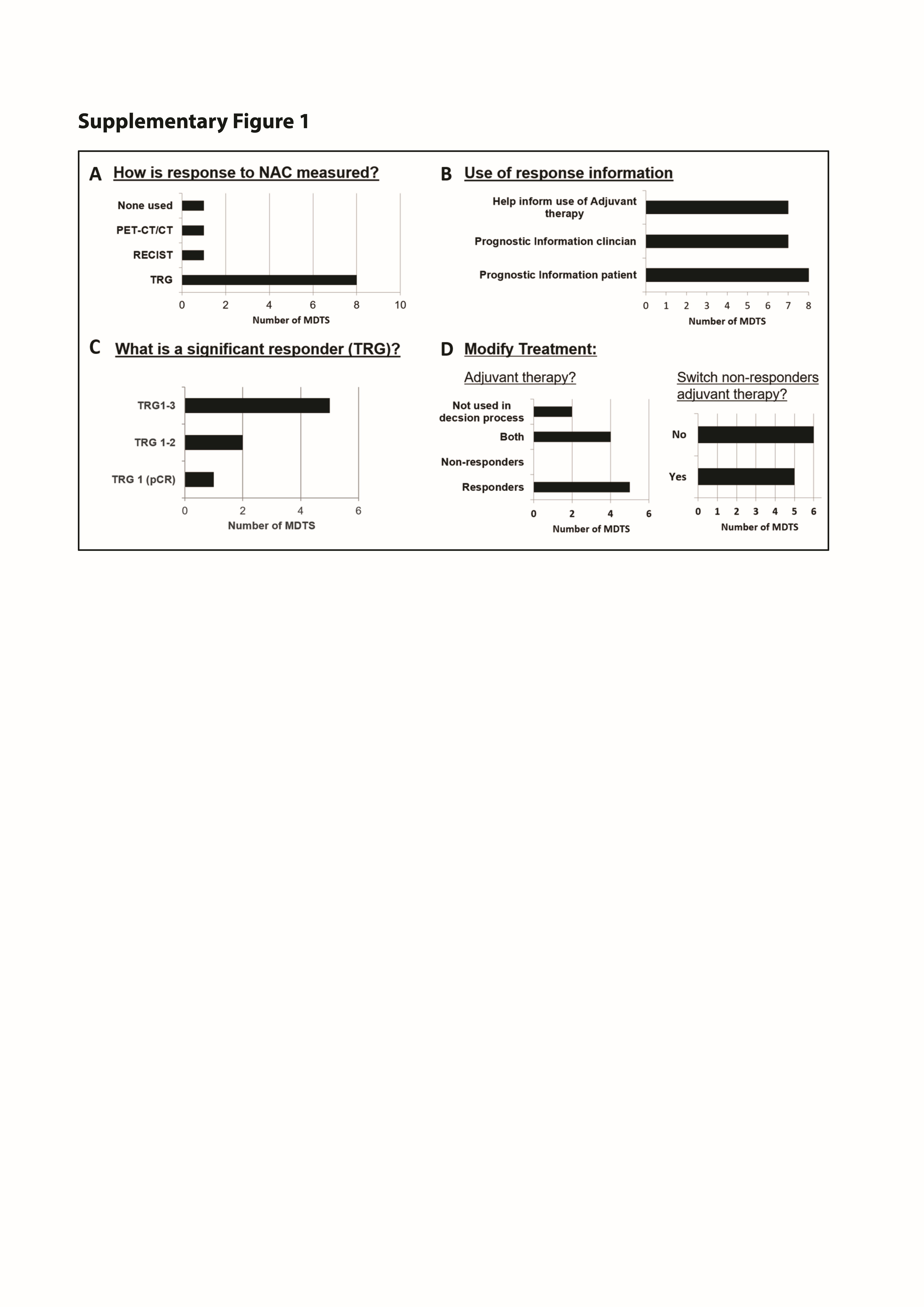


**Fig. S1** Responses to questionnaire sent to 11 UK cancer centres to determine current use of pathological response information in clinical decision-making


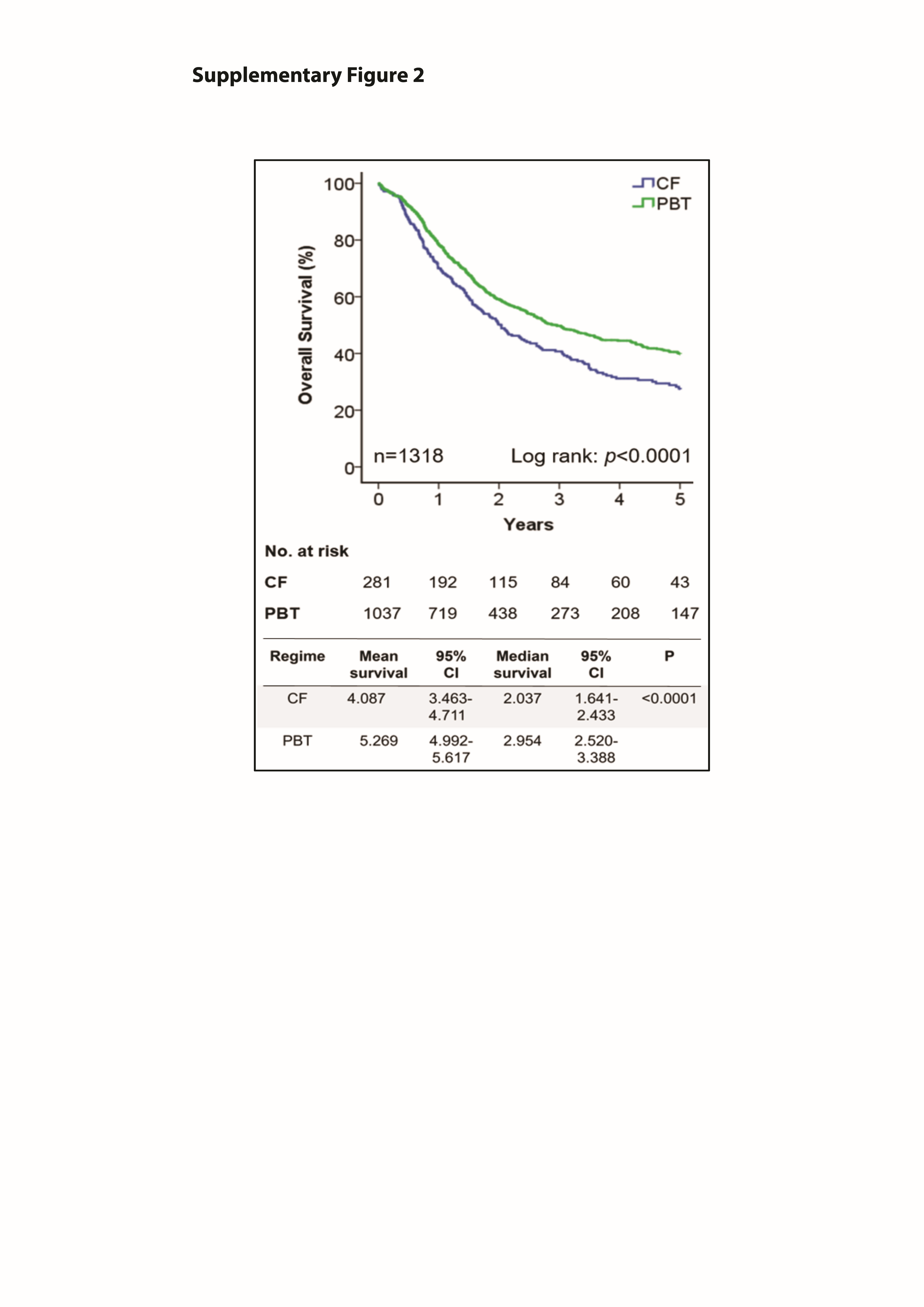


**Fig. S2** Kaplan–Meier curves for patients treated with cisplatin and 5-fluorouracil or platinum-based triplet chemotherapy

**Table S1** Resection margin involvement in relation to tumour regression grade


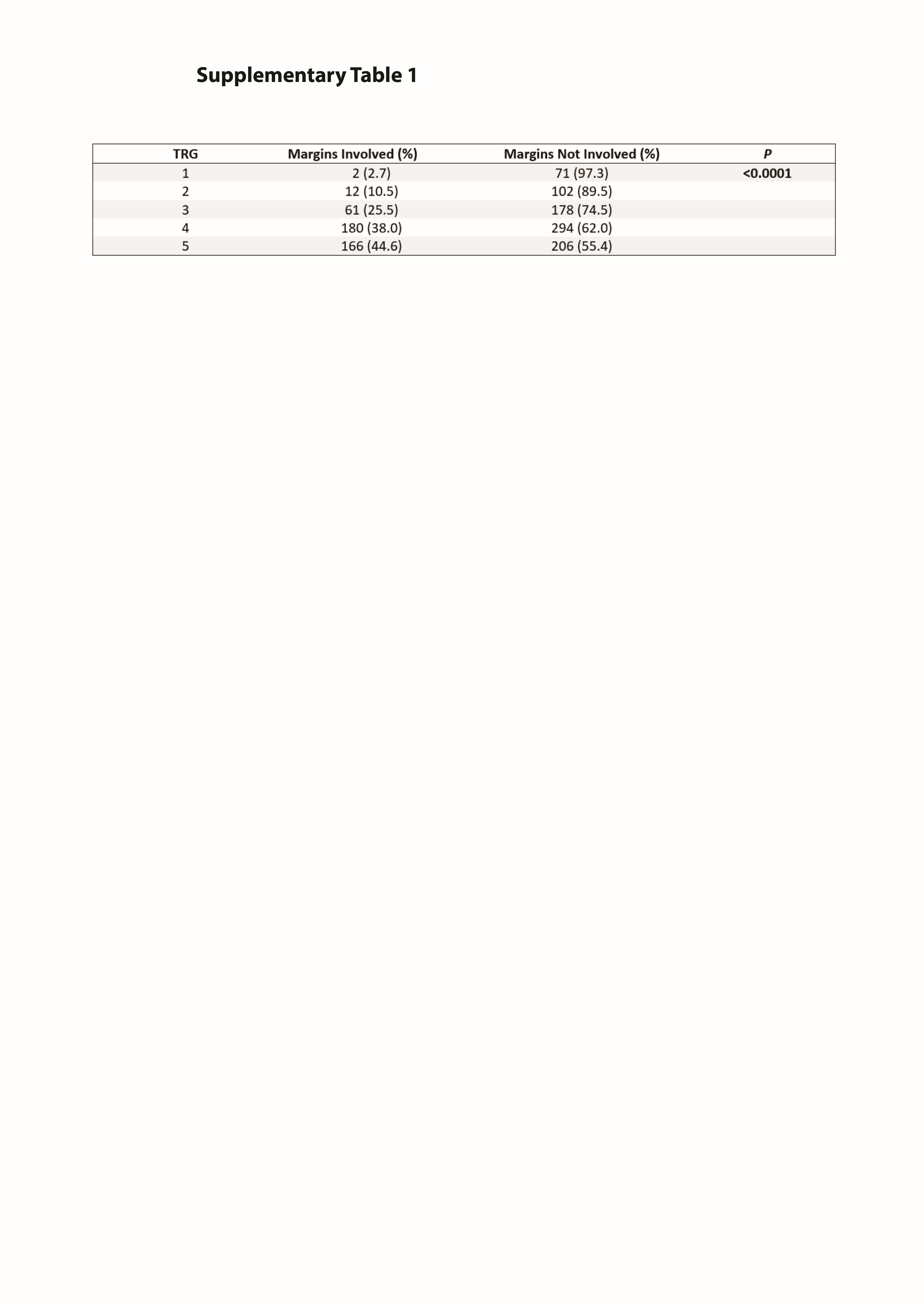


**Table S2** Effect of chemotherapy regimen on tumour regression grade, lymph node downstaging and resection margins


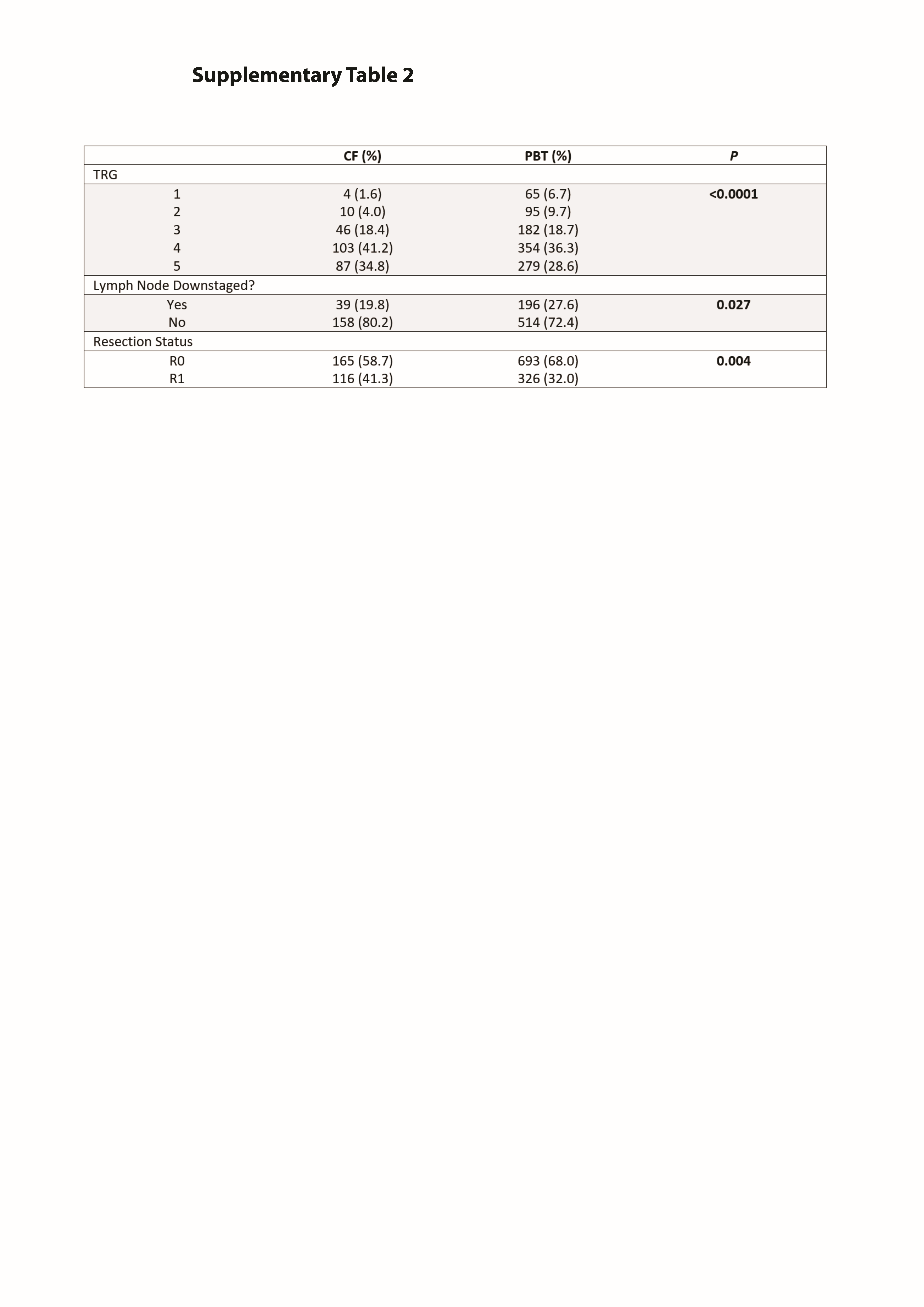

Supplement: bjs10627-0001-AppendixS1 — Appendix S1 Site-specific questionnaire [file bjs10627-0001-appendixs1.doc]
