# Supplementary figures and images for: Multicentre cohort study to define and validate pathological assessment of response to neoadjuvant therapy in oesophagogastric adenocarcinoma
Source: Br J Surg. 2017 Sep 25;104(13):1816–28. doi: 10.1002/bjs.10627 (PMC5725679; doi:10.1002/bjs.10627)

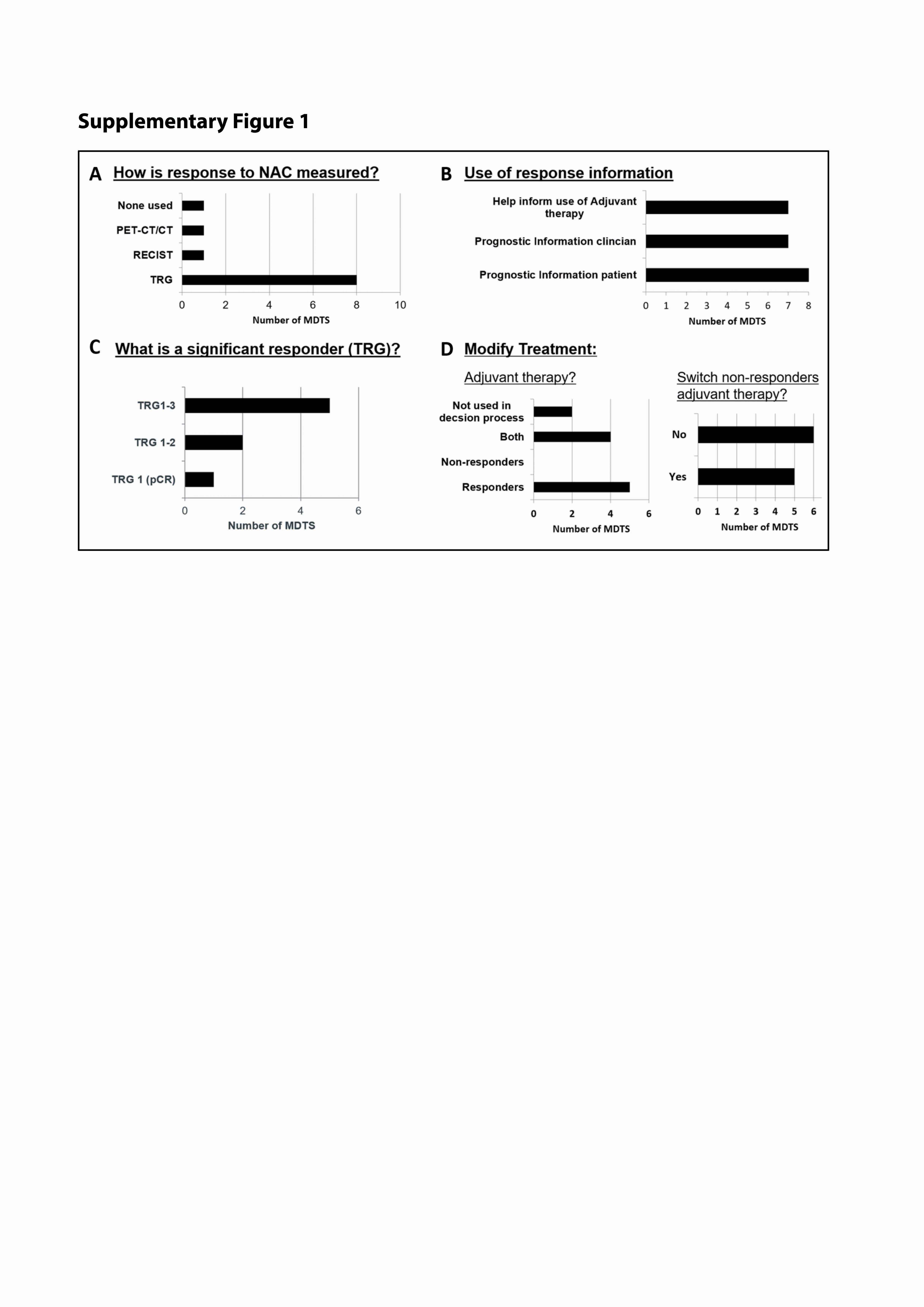

Supplement: bjs10627-0002-FigureS1 — Fig. S1 Responses to questionnaire sent to 11 UK cancer centres to determine current use of pathological response information in clinical decision-making [file bjs10627-0002-figures1.jpeg]

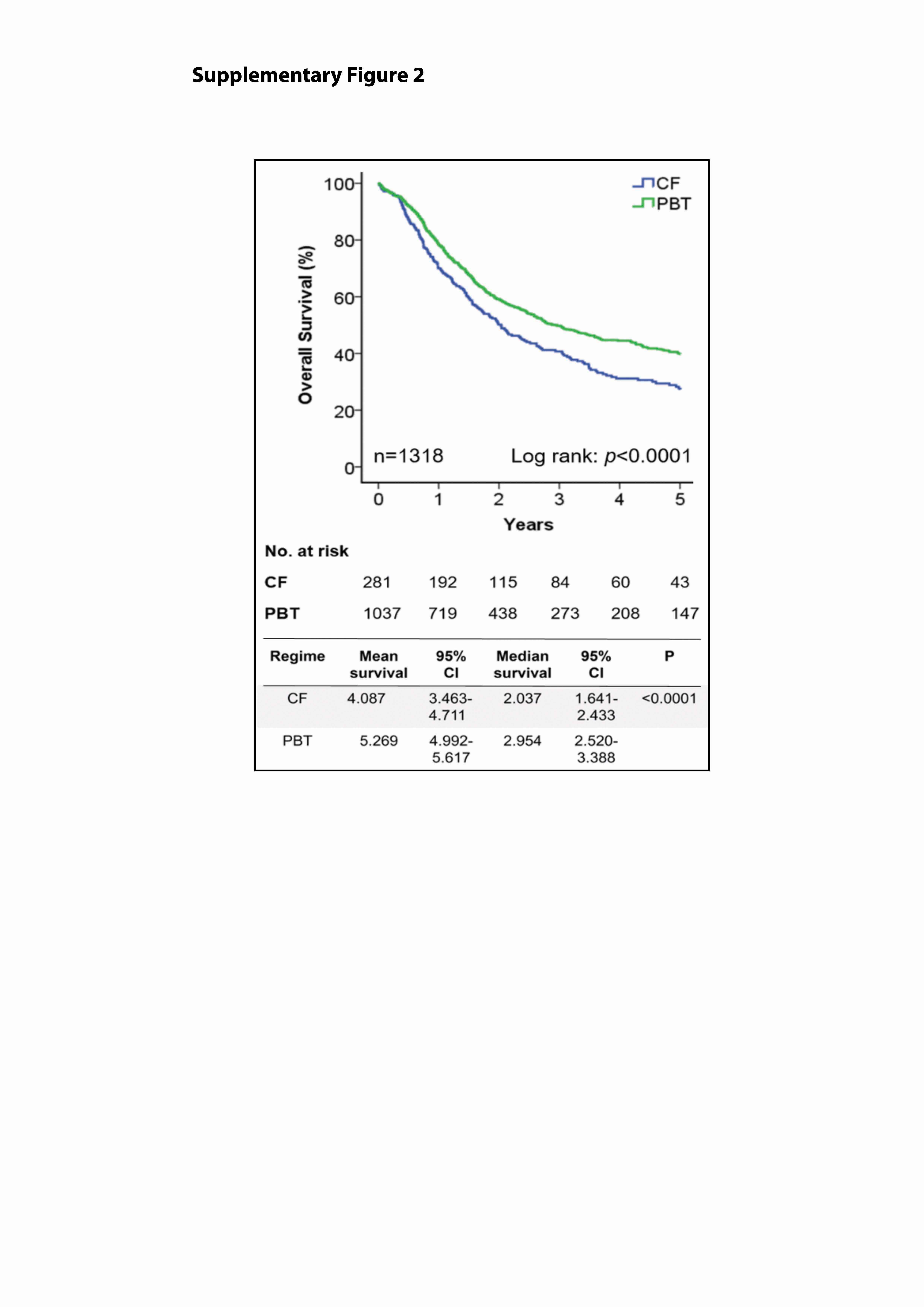

Supplement: bjs10627-0003-FigureS2 — Fig. S2 Kaplan–Meier curves for patients treated with cisplatin and 5-fluorouracil or platinum-based triplet chemotherapy [file bjs10627-0003-figures2.jpeg]

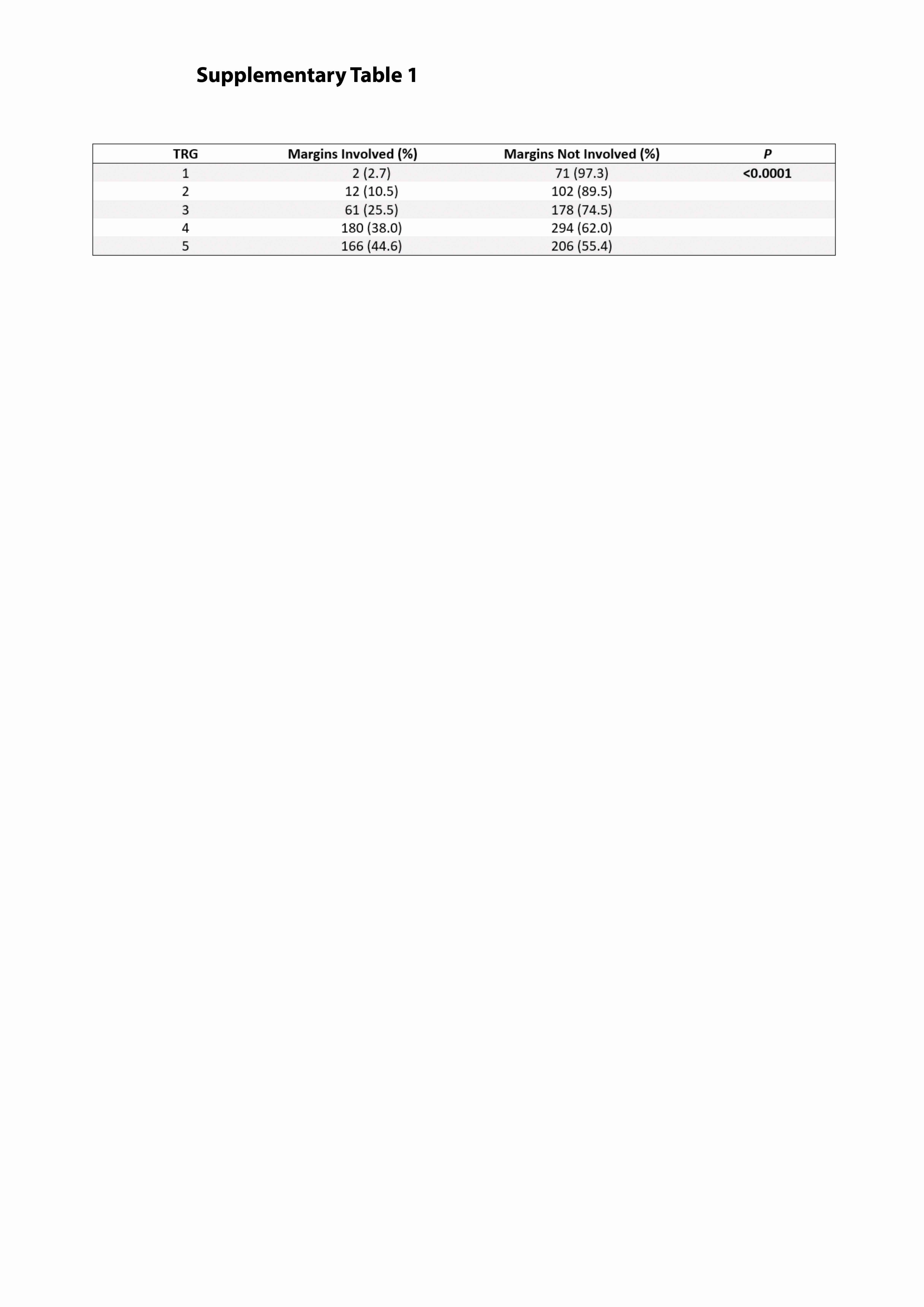

Supplement: bjs10627-0004-TableS1 — Table S1 Resection margin involvement in relation to tumour regression grade (Word document) [file bjs10627-0004-tables1.jpeg]

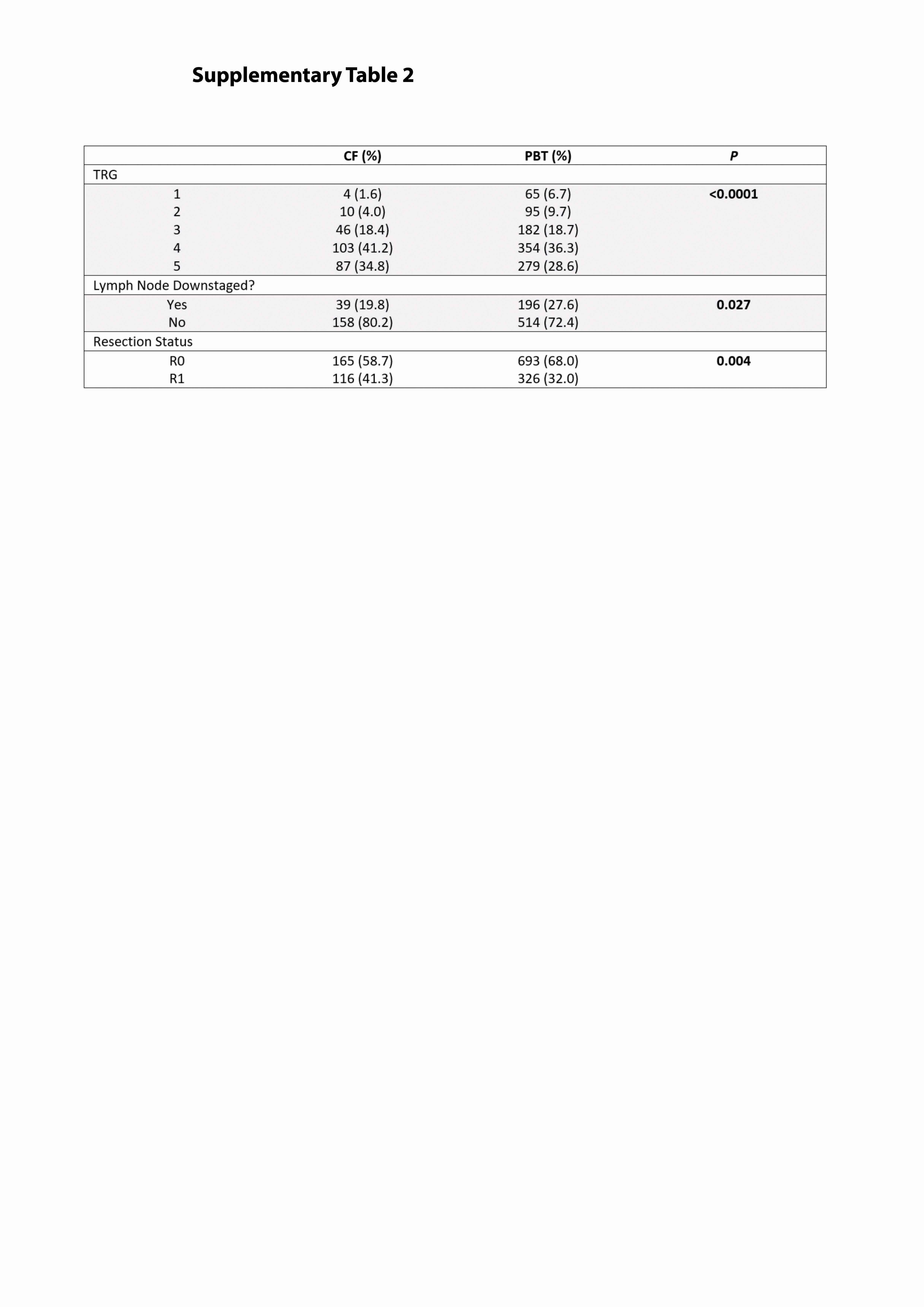

Supplement: bjs10627-0005-TableS2 — Table S2 Effect of chemotherapy regimen on tumour regression grade, lymph node downstaging and resection margins [file bjs10627-0005-tables2.jpeg]
